# Skull Base Tumors: Therapeutic Challenges and Multi-Disciplinary Care

**DOI:** 10.3390/cancers16030620

**Published:** 2024-01-31

**Authors:** Garret Choby

**Affiliations:** Center for Cranial Base Surgery, Departments of Otolaryngology and Neurological Surgery, University of Pittsburgh Medical Center, Pittsburgh, PA 15213, USA; chobygw2@upmc.edu

This special edition of *Cancers*, focusing on skull base tumors, highlights the unique pathologies affecting this anatomic location, as well as the multidisciplinary care necessary to treat these tumors.

Clival chordomas are rare but aggressive cranial base tumors that arise from notochordal remnants [1,2,3] in the clivus and pose significant treatment challenges due to their difficult anatomic location and close proximity to vital structures, including the basilar artery, internal carotid arteries, and several cranial nerves [4,5,6,7,8,9]. Recurrence is common [10,11,12,13,14]. Noya et al. report their institutional experience over a 20-year period, as well as a complimentary systematic review of the literature [15]. They report an overall survival (OS) of 49.9% at 10 years, with a progression-free survival (PFS) of 26.6% at 10 years. Their work demonstrated improved OS in younger patients, tumors with Ki67 < 5%, and with adjuvant radiotherapy. Tubin et al. highlight their experience with proton or carbon ion therapy for clival chordomas [16]. No difference in outcomes was noted between the radiotherapy modalities. Long-term control was improved in patients with a smaller targeted tumor volume, suggesting the importance of maximal safe resection prior to radiotherapy. Finally, Hong et al. examined a series of 58 clival chordoma patients at a single institution [17]. Compared to a historically treated cohort prior to 2013, those patients treated in the modern era had improved gross total resection rates, reduced rates of post-operative cranial nerve deficits, and improved PFS.

Locoregionally advanced and recurrent sinonasal malignancies are also highlighted. Sinonasal malignancies have traditionally had poor survival outcomes [18,19,20,21,22,23,24,25] with significant quality of life burden [26,27,28,29,30]; treatment is often dictated by the extent of the tumor and histologic subtype [24,31,32,33,34]. Melder and Geltzeiler present a comprehensive review of induction chemotherapy (IC) for locoregionally advanced squamous cell carcinomas (SCCA) and sinonasal undifferentiated carcinomas (SNUC) [35]. They report IC as a vital component of an organ-preservation approach to T4 SCCAs with orbital involvement. IC is also a vital upfront strategy for the treatment of SNUCs and can serve as a branchpoint for subsequent treatment, with a positive response to IC suggesting a subsequent positive response to consolidated chemoradiotherapy. Salvage therapy for recurrent esthesioneuroblastomas (ENB) was investigated by Ni et al. [36]. Amongst a large cohort of 143 ENB patients, 64 patients experienced recurrence. The time to recurrence was shorter in those with higher Hyams tumor grade. After salvage therapy, the subsequent 5-year OS was 63%, suggesting that salvage therapy can be effective for ENB.

A number of other important topics are also investigated, including anatomical considerations in surgical approaches to the anterior cranial base [37], quality of life considerations following treatment of cranial base tumors [38], management of pituitary tumors, and pseudoprogression of vestibular schwannomas following stereotactic radiosurgery [39]. In conclusion, the collection of articles in the Special Issue “Skull Base Tumors” has made a substantial contribution to the literature in regard to management approaches and outcomes with these challenging groups of tumors.
